# Diagnostic value of the combination of DAPK methylation in urinary sediment and B ultrasound for recurrent urinary bladder cancer

**DOI:** 10.1186/s12957-023-03103-9

**Published:** 2023-08-26

**Authors:** Dawen Wang, Zhilei Qiu, Changli Wu

**Affiliations:** 1 Department of Urology, The Second Hospital of Tianjin Medical University, No.23, Pingjiang Road, Hexi District, Tianjin, 300211 China; 2 Department of Urology, Qingdao Municipal Hospital, Qingdao, 266071 Shandong China

**Keywords:** DAPK, B ultrasound in bladder, Urinary sediment, Urinary bladder cancer, Follow-up

## Abstract

**Background:**

Urinary bladder cancer (UBC) is the most common malignancy affecting the urinary system. This study aimed to investigate the diagnostic value of combining DAPK methylation in urinary sediment and B ultrasound in the detection of recurrent UBC.

**Methods:**

A total of 1021 cases with primary UBC who underwent electrocision of bladder tumor through urethra were included in this study and followed up. Various parameters including B ultrasound, DAPK methylation in urinary sediment, examination of exfoliated cells in the urine, and cystoscopy were performed. The data collected was analyzed using the Kappa test, and receiver operating characteristic (ROC) curve was constructed to assess the diagnostic role in recurrent UBC.

**Results:**

Among the 1021 patients, 115 patients experienced recurrence confirmed by cystoscopy and biopsy within two years and were excluded from the study, resulting in an effective sample size of 906 primary UBC cases. The results of cystoscopy showed agreement with B ultrasound (Kappa = 0.785, *P* < 0.05), as well as with DAPK methylation in urinary sediment, and the combination of B ultrasound and DAPK methylation (Kappa = 0.517, *P* < 0.05, Kappa = 0.593, *P* < 0.05). The combination of B ultrasound with DAPK methylation yielded an area under the curve of 0.922, with a sensitivity of 92.86%, specificity of 91.63%, and a negative predictive value of 99.4%, suggesting that a negative result indicates a low risk of recurrence.

**Conclusion:**

The combination of DAPK methylation in urinary sediment with B ultrasound demonstrates high diagnostic performance for recurrent UBC.

## Introduction

Urinary bladder cancer (UBC) is a prevalent cancer worldwide, with a significant number of new cases diagnosed each year. It ranks among the most common cancer globally, with an estimated 430,000 new cases reported in 2012 [1, 2]. The incidence of UBC is higher in individuals over the age of 65 years, particularly those older than 70, where it is seven to ten times more common [3]. Major risk factors for UBC include exposure to carcinogens, especially tobacco smoking, and the male sex, as the incidence of UBC in males is three to four times higher than in females [4]. Unfortunately, UBC often goes undiagnosed until patients present with visible blood in the urine (macroscopic hematuria), leading to a higher risk of mortality compared to those diagnosed at earlier stages [5, 6]. Therefore, improving early detection methods for UBC is crucial for the well-being of patients.

Currently, the most common approaches for UBC detection include cystoscopy, urinary cytology, B ultrasound in the bladder, and the use of urinary biomarkers as alternatives or complementary methods to cystoscopy [7]. Cystoscopy is considered the gold standard but is invasive and relatively expensive. Urinary cytology, a non-invasive method, is widely used but has limited effectiveness, particularly for low-grade UBC [8, 9]. Ultrasonography has shown promise as a follow-up tool for low-risk UBC, serving as an alternative to cystoscopy [10]. Previous studies have also indicated that UBC patients exhibit significantly increased methylation of the DAPK promoter compared to normal controls, suggesting the potential of DAPK methylation as a biomarker for UBC detection [11].

Despite the primary reliance on cystoscopy and urinary cytology for UBC diagnosis, these methods have limitations which have prompted the exploration of alternative, minimally invasive, approaches. In this study, we aimed to investigate the diagnostic value of detecting DAPK methylation in urinary sediment, B ultrasound in the bladder, and urinary cytology as follow-up methods for UBC patients, using cystoscopy results as the benchmark.

## Material and method

### Ethical statement

The study was conducted in accordance with the protocols approved by the Second Hospital of Tianjin Medical University (No: 2021098). Informed signed consents were obtained from all participants prior to specimen collection.

### Subjects

A total of 1021 patients with UBC who underwent surgery at China-Japan Union Hospital between June 2014 and June 2016 were enrolled in the study. The eligibility criteria included patients with primary UBC and complete medical records. Patients with complications such as kidney and liver diseases, other urinary system diseases (including renal pelvic carcinoma, ureteral carcinoma, urinary tract carcinoma, and renal carcinoma), other tumors, or unclear pathological diagnosis were excluded. All included patients underwent urinary bladder irrigation chemotherapy at fixed periods. Patients at high risk underwent secondary surgery approximately 4 to 6 weeks after primary surgery.

### Follow-up

Regular follow-up was conducted for a period of three months within 2 years after the surgery. The follow-up assessments included DAPK methylation in urinary sediment, B ultrasound in the bladder, detection of exfoliated cells in the urine, and cystoscopic observation. Each test was performed by a physician who was blinded to the results of other detections. The follow-up period continued for two years after primary surgery or until radical cystectomy was performed in case of invasive UBC.

### DAPK methylation in urinary sediment

A total of 50 ml of urine samples were collected on the morning of the day of cystoscopy. The samples were centrifuged at 2000 rpm/min for 10 min at 4 °C. The resulting urine sediment was rinsed twice with PBS and dissolved in 200 ul of pure water, and stored in a refrigerator at − 80 °C for further use. Tissue and genomic DNA kits from Tiangen Bio-technology Ltd., Co (Beijing, China) were used to extract the DNA content. The purity and content of the extracted DNA were assessed using an ultraviolet spectrophotometer (A260/A280 > 1.8), and the DNA was preserved in a 1.5-ml centrifuge tube at − 20 °C. The extracted genomic DNA was modified using the EpiTect Bisulfite kits (Qiagen Corporation, Germany) with hydrosulphite. The modified genomic DNA was stored at -80 °C and used within 1 month. The conversion efficiency of the kit was confirmed to be 99% or above by adding spike-in DNA to detect the transformation of cytosine in non-CpG sequences. The modified genomic DNA were subjected to methylated specific polymerase chain reaction (PCR) and non-methylated specific PCR. The upstream and downstream primers for methylation were: 5′-GGATAGTCGGATCGAGTTAACGTC-3′ and 5′-CCCTCCCAAACGCCGA-3′, respectively, resulting in a 98 bp amplification product. The upstream and downstream primers for non-methylation were: 5′-GGAGGATAGTTGGATTGAGTTAATGTT-3′ and 5′-CAAATCCCTCCCAAACACCAA-3′, respectively, resulting in a 106-bp amplification product. All primers were synthesized by Shanghai Sangon Biotech. The PCR reaction system (25 μl) consisted of 2 ul of modified DNA, 0.5 μl of upstream primer, 0.5 μl of downstream primer, 2.5 μl of 10 × PCR buffer, 0.5 ul of Taq, and 19 μl of ultrapure water. The PCR amplification conditions were as follows: pre-denaturation at 94 °C for 5 min, followed by 40 cycles of denaturation at 94 °C for 60 s, annealing at 54 °C for 30 s, extension at 72 °C for 30 s, and a final extension at 72 °C for 10 min. Two blank controls were included in each reaction for quality control, and two parallel samples were prepared for each group. The PCR products were separated by electrophoresis on a 2% Sepharose gel and visualized under a UV lamp (ultraviolet lamp) for photography.

### B ultrasound in the bladder

Prior to undergoing transabdominal B ultrasound in the bladder, patients were instructed to hold their urine. The ultrasound examination was performed by an appointed associate chief physician at the B ultrasound department.

### Cytology observation

Cytology observation in urine was conducted routinely, with each patient required to provide urine samples for three consecutive days. The cells in the urine samples were stained using the Papanicolaou (Pap) staining method. Cytology results were considered positive if tumor cells were detected in the urine, and negative in no tumor cells were observed.

### Cystoscopy

Cystoscopy procedures were performed by an appointed and experienced physician. Patients were positioned in the lithotomy position and received either local anesthesia or intravenous anesthesia. The cystoscopy was inserted at a 70° angle to examine the bladder for tumor recurrence. Any unidentified lesions were biopsied and sent for pathological examination.

### Statistical analysis

Data analysis and graphing were performed using SPSS 22.0 (IBM Corp., Armonk, NY, USA), GraphPad Prism 8 (GraphPad Software, San Diego, CA, USA), and Medcalc® version 15.0 (Medcalc Software Ltd, Ostend, Belgium) software. Enumeration data were presented as cases. The sensitivity, specificity, Youden index (YI), positive predictive values, negative predictive values of results of B ultrasound in the bladder, DAPK methylation examination in urinary sediment, and urinary cytology observation were compared to the results of cystoscopy, which served as the benchmark. Pairwise chi-square (*x*^2^) and Kappa tests were used for comparison. The area under the ROC curve was analyzed using MedCalc’s “Comparison of ROC curve” analysis. The results of B ultrasound in the bladder and DAPK methylation were combined, considering either of the two results being positive as a positive result, and both of the two methods being negative as a negative result. A *p* value of less than 0.05 was considered statistically significant.

## Results

### Identification of DAPK methylation in urinary sediment

**Fig. 1:**
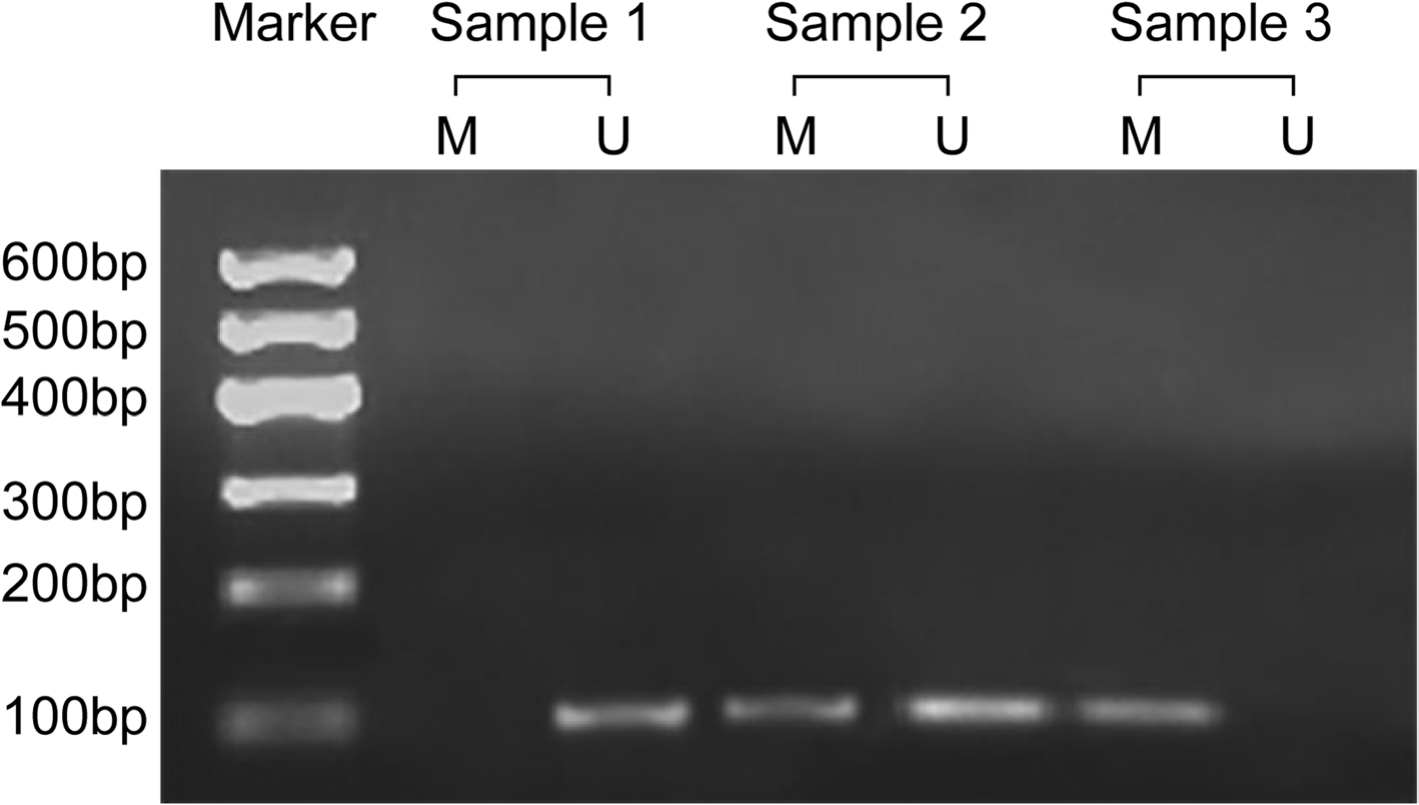
Identification for DAPK methylation in urinary sediment. Note: M represents the methylated band, U represents the non-methylated band. Sample 1 indicates non-methylation, sample 2 indicates partial methylation, and sample 3 indicates complete methylation

To identify DAPK methylation in urinary sediment, the collected samples underwent both methylated specific PCR and non-methylated specific PCR. If the amplified products were obtained only with non-methylated primer, the samples were considered to have complete methylation in the DAPK promoter region. Samples showing amplified products for both methylated primer and non-methylated primer were considered partially methylated. Non-methylated products indicated samples without methylation. Samples with complete methylation or partial methylation were considered positive for DAPK methylation (Fig. 1).

### Follow-up results

Among the initial 1021 patients, a total of 115 patients did not complete the 2-year follow-up due to various reasons. Therefore, the effective number of patients followed up was 906. B ultrasound results revealed that 55 patients showed space-occupying lesions in the bladder. Among them, 50 patients were confirmed to have tumor recurrence based on cystoscopy and biopsy, with a minimum tumor size of 0.6 cm. The remaining 5 patients were diagnosed with cystitis. DAPK methylation in urinary sediment identified 118 patients as positive and 788 patients as negative. Furthermore, the presence of exfoliated cells in urine indicated tumor recurrence in 27 patients. The combination of B ultrasound in the bladder and DAPK methylation in urinary sediment identified 135 patients as positive (Table 1).

**Table 1:** Identification of recurrent UBC by B ultrasound in the bladder, DAPK methylation in urinary sediment, and observation in exfoliated cells in urine

	Cystoscopy	B ultrasound in the bladder		DAPK methylation in urinary sediment		Exfoliated cells in urine		Combination of DAPK methylation and B ultrasound in bladder	
		Positive	Negative	Positive	Negative	Positive	Negative	Positive	Negative
Positive	70	50	20	53	17	27	43	65	5
Negative	836	5	831	65	771	0	836	70	766
Total	906	55	851	118	788	27	879	135	771

Combination of DAPK methylation and B ultrasound in the bladder, either of the inspection results being positive shall be considered as positive for combination inspection and both of the two inspections being negative shall be considered as negative for combination inspection; *UBC*, urinary bladder cancer

### Comparisons of UBC Recurrence Identification

A comparison between cystoscopy and B ultrasound in the bladder showed a Kappa value of 0.785, indicating a high level of agreement between the two methods (Kappa > 0.75). The *p* value was less than 0.05, further confirming their significant consistency. Comparing cystoscopy with DAPK methylation in urinary sediment, the Kappa test showed a value of 0.517, indicating a lower level of consistency (Kappa of 0.4 ~ 0.75). However, the p-value was less than 0.05, indicating some degree of agreement between the two methods. Comparison between cystoscopy and the observation of exfoliated cells in urine yielded a Kappa value of 0.537, with a *p*-value greater than 0.05, suggesting a coincidental consistency without statistical significance. Additionally, the comparison between cystoscopy and combined detection resulted in a Kappa value of 0.593, with a *p*-value less than 0.05, indicating a general consistency between these two methods (Table 2).

**Table 2:** Analysis on identification of recurrent UBC by cystoscopy, B ultrasound in the bladder, DAPK methylation in urinary sediment, and observation in exfoliated cells in urine

		B ultrasound in the bladder	DAPK methylation in urinary sediment	Exfoliated cells in urine	Combination of DAPK methylation and B ultrasound in bladder
Cystoscopy	Kappa	0.785	0.517	0.537	0.593
	*P*	0.041	0.046	0.061	0.041

*UBC* urinary bladder cancer

### Evaluation of diagnosing recurrent UBC

**Fig. 2:**
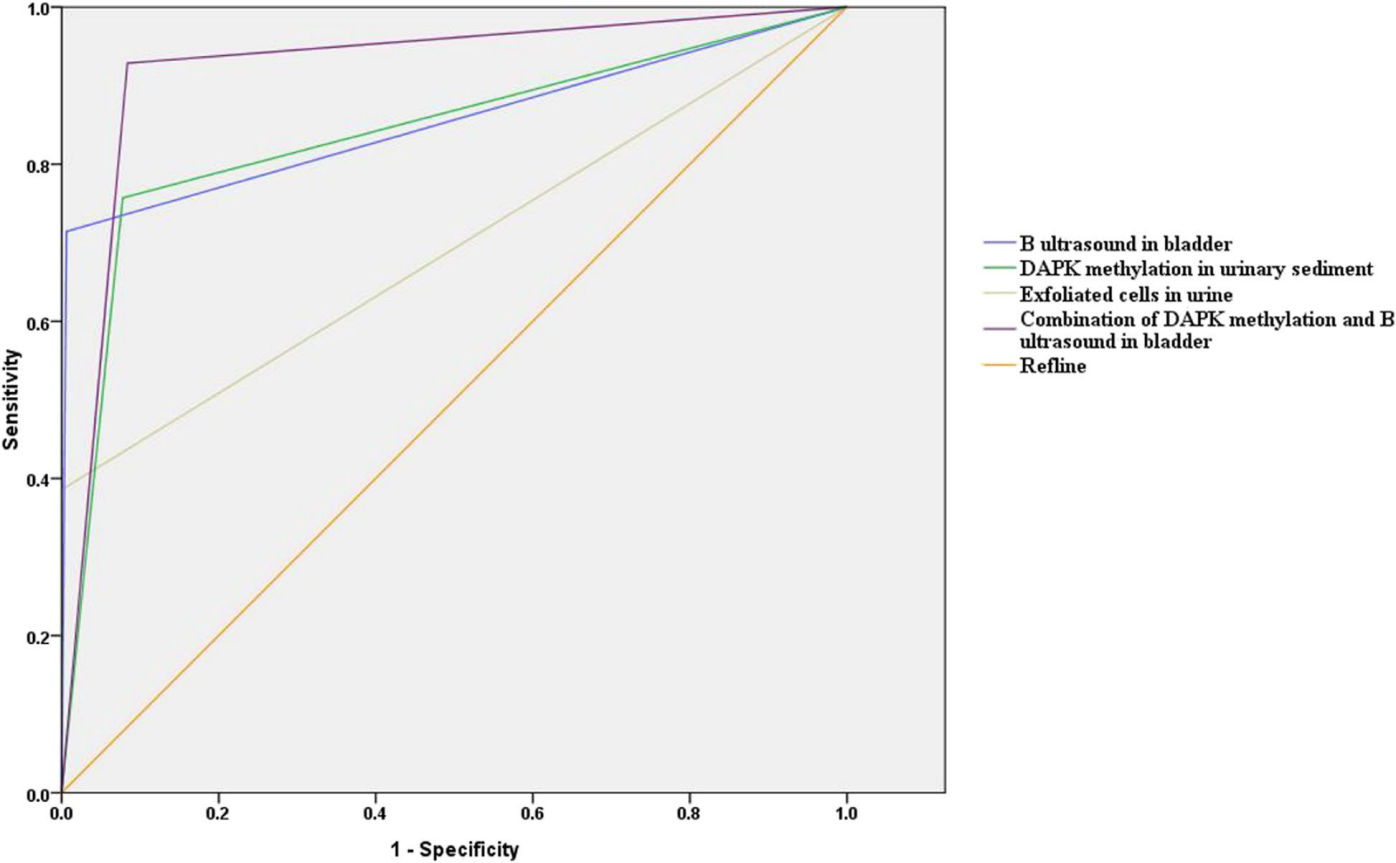
ROC curves for diagnosing recurrent UBC by B ultrasound in the bladder, DAPK methylation in urinary sediment, and observation in exfoliated cells in urine

To evaluate the methods for diagnosing recurrent UBC, a receiver operating characteristic (ROC) curve was plotted, and the area under the curve (AUC), sensitivity, specificity, and YI (YI = sensitivity + specificity − 1) were calculated (Table 3 and Fig. 2). The combined detection method had an AUC of 0.922, sensitivity of 92.86%, specificity of 91.63%, and YI of 0.845. The analysis of the ROS curve differences showed that combined detection outperformed the other methods (*p* = 0.0066, *p* = 0.0003, *p* < 0.0001). The positive predictive value of combined detection was 48.15% and the negative predictive value was 99.35% when comparing the two values of all methods. This highlights the significance of combined detection in excluding a diagnosis.

**Table 3:** Evaluation on diagnosing recurrent UBC by B ultrasound in the bladder, DAPK methylation in urinary sediment, and observation in exfoliated cells in urine

	AUC	Sensitivity (%)	Specificity (%)	Confidence interval	Youden index	Positive predict value (%)	Negative predict value (%)
B ultrasound in the bladder	0.854	71.43	99.04	0.790–0.918	0.708	90.91	97.65
DAPK methylation in urinary sediment	0.84	75.71	92.22	0.780–0.900	0.679	44.92	97.84
Exfoliated cells in urine	0.693	38.57	100	0.614–0.772	0.386	100	95.11
Combination of DAPK methylation and B ultrasound in bladder	0.922	92.86	91.63	0.886–0.959	0.845	48.15	99.35

Youden index = sensitivity + specificity − 1; positive predict value = cases of true positive/(cases of true positive + cases of false positive) × 100%; negative predict value = cases of true negative/(cases of true negative + cases of false negative) × 100%; *UBC*, urinary bladder cancer, *AUC*, area under curve

## Discussion

UBC is the most prevalent malignancy of the urinary tract in both males and females, with cystoscopy being the gold-standard approach for detection and monitoring of both primary tumor and follow-up after resection [12]. However, cystoscopy has limitations, including a false negative rate of 10–40%, limiting its clinical efficacy [13]. In this study, we included 1021 patients diagnosed with primary UBC and analyzed their 2-year follow-up data to investigate the optimal diagnostic tool for diagnosing recurrent UBC. We compared the diagnostic accuracy of B ultrasound in the bladder, DAPK methylation in urinary sediment, urinary cytology, and combination of B ultrasound with DAPK methylation. Our findings suggest that the combination of B ultrasound and DAPK methylation in urinary sediment exhibits high performance in diagnosing recurrent UBC, providing a potentially more reliable tool for diagnosis.

Although there are limited studies on the methylation status of DAPK in UBC, existing reports confirm the differential expression of DAPK in urine [11, 14–17]. Our initial findings demonstrated a high consistency between B ultrasound in the bladder and cystoscopy, suggesting that ultrasonography may be a reliable tool for diagnosing UBC. The AUC of ultrasonography was found to be 0.854, with a sensitivity of 71.43% and a specificity of 99.04%. These results align with prior studies that highlight the advancements made in transabdominal ultrasonography for visualizing intraluminal filling defects in the bladder [18]. Moreover, previous research has shown the potential of DAPK gene methylation as a urine biomarker for bladder cancer diagnosis [19]. While ultrasonography lags behind cystoscopy in accuracy, considering its value as a surveillance tool, it can be a useful adjunct for UBC screening, taking into account operator skill, amount of abdominal fat, and bladder distension during the procedure.

Methylation of tumor suppressor gene promoters is widely-known to lead to transcriptional inactivation and is implicated in tumorigenesis [11]. The DAPK gene, a tumor suppressor gene, plays a critical role in inducing cell proliferation suppression and apoptosis [15, 20]. Notably, Jablonowski et al. reported a 64.3% DAPK methylation rate in UBC patients [15]. In our study, we detected DAPK promoter methylation in urinary sediment of UBC patients, and the ROC curve showed an AUC of 0.840, sensitivity of 75.71%, specificity of 92.22%, and YI of 0.679 for DAPK methylation. Furthermore, when combining ultrasonography with DAPK methylation detection in urinary sediment, we observed the highest AUC, sensitivity, specificity, and YI among all approaches, with values of 0.922, 92.86%, 91.63%, and 0.845, respectively. These findings support the superiority of combining ultrasonography with DAPK methylation detection over other single approaches. Additionally, the positive predictive value of combined detection was 48.15%, lower than other methods, suggesting its significance in excluding diagnosis.

Subsequently, we evaluated the diagnostic performance of urinary cytology in UBC and found it to be significantly inferior compared to ultrasonography and DAPK methylation detection. The ROC curve analysis revealed an AUC of only 0.693 for urinary cytology, with a sensitivity of 38.57%. However, it exhibited a high specificity of 100%. These results align with previous observations that cytology, despite its lower sensitivity, remains the preferred tumor marker for bladder tumors due to its superior specificity [21].

## Conclusion

In conclusion, our study demonstrates that the combination of DAPK methylation in urinary sediment and B ultrasound in the bladder offers improved accuracy in the diagnosis of UBC compared to individual approaches. This suggests that the combined detection of DAPK methylation and B ultrasound could potentially reduce or even replace the need for cystoscopy in clinical practice for diagnosing bladder cancer. However, our study did not analyze the underlying factors influencing the positivity of DAPK methylation and B ultrasound. Future research should focus on evaluating the screening and diagnostic tools for bladder cancer using the combined detection of DAPK methylation and B ultrasound, taking into consideration factors such as histological grade, tumor stage, grade, muscle invasiveness, and racial/ethnic disparities. Furthermore, larger studies with diverse patient populations are needed to validate our findings and ensure the reliability of clinical research in this field.

## Data Availability

The datasets generated during and/or analyzed during the current study are available from the corresponding author on reasonable request.
